# Effect of Nocturnal Hemodialysis versus Conventional Hemodialysis on End-Stage Renal Disease: A Meta-Analysis and Systematic Review

**DOI:** 10.1371/journal.pone.0169203

**Published:** 2017-01-20

**Authors:** Fangjie Liu, Yiting Sun, Tianhua Xu, Li Sun, Linlin Liu, Wei Sun, Xin Feng, Jianfei Ma, Lining Wang, Li Yao

**Affiliations:** 1 Department of Nephrology, The First Hospital of China Medical University, Shenyang, Liaoning, China; 2 Department of Clinical Medicine, China Medical University, Shenyang, Liaoning, China; 3 Department of General Surgery, Shengjing Hospital of China Medical University, Shenyang, Liaoning, China; 4 Blood Purification Center, Liaoning Electric Power Center Hospital, Shenyang, Liaoning, China; Kaohsiung Medical University Hospital, TAIWAN

## Abstract

**Objectives:**

The purpose of this study is to assess the efficacy and safety of nocturnal hemodialysis on end-stage renal disease (ESRD) patients.

**Methods:**

We searched Medline, EmBase, and the Cochrance Central Register of Controlled Trials for studies up to January 2016. Analysis was done to compare variant outcomes of different hemodialysis schedules, including mortality, cardiovascular-associated variables, uremia-associated variables, quality of life (QOL), side-effects, and drug usage.

**Results:**

We collected and analyzed the results of 28 studies involving 22,508 patients in our meta-analysis. The mortality results in this meta-analysis indicated that the nocturnal hemodialysis (NHD) group was not significantly different from conventional hemodialysis (CHD) group (Mortality: OR: 0.75; 95% confidence intervals (CIs): 0.52 to 1.10; p = 0.145), but the CHD group had significantly fewer number of hospitalizations than the NHD group (OR: 1.54; 95%CI: 1.32 to 1.79; p<0.001). NHD was superior to CHD for cardiovascular-associated (left ventricular hypertrophy [LVH]: SMD: -0.39; 95%CI: -0.68 to -0.10; p = 0.009, left ventricular hypertrophy index [LVHI]: SMD: -0.64; 95%CI: -0.83 to -0.46; p<0.001) and uremia-associated intervention results (Serum albumin: SMD: 0.89; 95%CI: 0.41 to 1.36; p<0.001). For the assessment of quality of life, NHD treatment significantly improved the patients’ QOL only for SF36-Physical Components Summary (SMD: 0.43; 95%CI: 0.26 to 0.60; p<0.001). NHD intervention was relatively better than CHD for anti-hypertensive drug usage (SMD: -0.48; 95%CI: -0.91 to -0.05; p = 0.005), and there was no difference between groups in our side-effects assessment.

**Conclusion:**

NHD and CHD performed similarly in terms of ESRD patients’ mortality and side-effects. NHD was superior to CHD for cardiovascular-associated and uremia-associated results, QOL, and drug usage; for number of hospitalizations, CHD was relatively better than NHD.

## Introduction

End-stage renal disease (ESRD) is a chronic and progressive decline in kidney function, which will eventually lead to uremia and death if it is not treated properly [1]. However, with a progress of technology in past decades, the mortality have not improved significantly and exceeding 20% in chronic hemodialysis patients [2, 3]. Cardiovascular events are the main driving force for this high mortality. Therefore, there is a need for new methods to improve ESRD patients’ cardiovascular and mortality risk.

There are currently two main methods for treatment of ESRD patients. The first, renal transplantation, is a permanent method to cure ESRD patients, however, that means ESRD patients have an issue of having a proper kidney source, thus it has limited application [4, 5]. The second, hemodialysis, is applied worldwide but has a high risk of cardiovascular complications and significantly reduces the quality of life of patients [6, 7]. Nocturnal hemodialysis (NHD) is an important branch of hemodialysis [8, 9]. The schedule for nocturnal dialysis is 3–7 times per week, 7–8 hours every time. This approach extends the effective duration of dialysis without affecting the patient’s daytime activities making it more convenient as a method of treatment. This approach has been widely used in Canada; however, the clinical results still require further examination. Dialysis-related disease is defined as the complications caused by long-term dialysis on ESRD patients; cardiovascular disease is the leading cause of death for ESRD patients [10–13].

Previously, several systemic reviews analyzed the mortality, blood pressure, and urinary-related indexes of NHD for ESRD patients [14–16]. However, the qualities of included studies were relatively low and not comprehensive evaluated all relevant clinical outcomes. Our research is up to date with recently published research and analyzes the effects of NHD by mortality, cardiovascular-related variables, uremia-related variables, quality of life, side-effects, and drug usage to provide better insight in clinical choices for dialysis methods.

## Methods

### Search strategy and selection criteria

This review was conducted and reported according to the Preferred Reporting Items for Systematic Reviews and Meta-Analysis Statement [17] issued in 2009. Any studies that examined NHD versus conventional hemodialysis (CHD) on ESRD patients were eligible for inclusion in our study with no restrictions placed on language or publication status (published, or in press). We searched the Medline, EmBase, and Cochrane Library electronic databases for articles published through January 2016 and used “nocturnal”, “dialysis”, “hemodialysis”, and “controlled trials” as the search keywords. We also conducted manual searches of reference lists from all relevant original articles and reviews to identify additional eligible studies.

A literature search was undertaken independently by 2 authors and any inconsistencies were settled via group discussion. A study was eligible for inclusion if the following criteria were met: (1) the trial investigated nocturnal hemodialysis NHD versus conventional hemodialysis CHD; (2) all of patients included with ESRD; and (3) the outcomes variable included one of the following: mortality, cardiovascular-associated variables, uremia-associated variables, quality of life, side-effect, and drug usage. Case series, reviews, and editorials were excluded.

### Data collection and quality assessment

Two reviewers independently extracted all data with disagreements resolved in consultation with third-party investigators. The following items were extracted from the included articles: first author, publication year, country, location or data source, study design, sample size, disease status, mean age, gender proportion, mean duration of dialysis, Dialysis session, and reported outcomes. The outcome assessments included: mortality, cardiovascular-associated variables, uremia-associated variables, quality of life, side-effects, and drug usage. In analysis, the numerical changes between, before, and after dialysis of statistical indicators had priority to be adopted, if not, the dialysis numerical indicators after dialysis was adopted. In addition, the numerical units were adjusted for consistency, such as g/L and g/dL. Two reviewers independently assessed the quality of included studies according to the Cochrane risk of bias tool in the following six domains: selection, performance, detection, attrition, reporting and other bias [18].

### Statistical analysis

For our meta-analysis, we used the inverse variance method to pool continuous data and the Mantel-Haenszel method for dichotomous data; the results are presented as standardized mean difference (SMD) with 95% confidence intervals (CIs) and odds ratio (OR) with 95%CIs. The I^2^ statistic was calculated to evaluate the extent of variability attributable to statistical heterogeneity between trials. In the absence of statistical heterogeneity (I^2^≤50%), we used a fixed-effect model, otherwise we used a random-effect model for traditional meta-analysis [19]. To investigate the sources of heterogeneity, predefined subgroup analysis were performed: dialysis schedule and design bias. We assessed for publication bias using the Begg-Mazumdar [20] and Egger’s test [21]. A non-parametric “Trim and Fill” method of assessing publication bias was applied if needed [22]. All tests were two tailed, and a p value of less than 0.05 was deemed statistically significant. We analyzed the data using Review Manager (Version 5.3) and STATA (Version 12.0).

## Results

**Fig 1:**
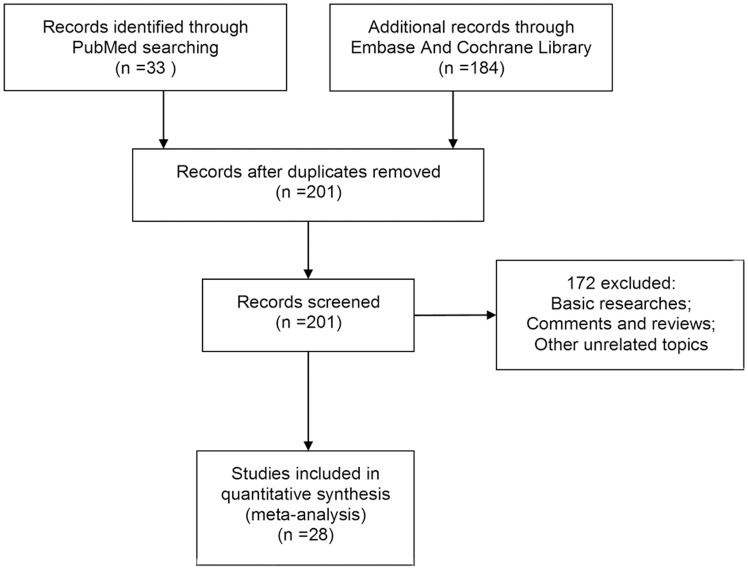
Flow diagram. PRISMA flow diagram.

Our research returned 201 results after removing duplicates, from which we collected 28 trials in our meta-analysis (Fig 1). After a full text review, the reasons for exclusion of literature included non-controlled trials, other intervention interference, other similar diseases, and lack of desired outcome assessments. The general characteristics of the included studies are presented in Table 1. In this research, included studies were mainly published in Canada, China, the United States, the United Kingdom, Australia, and Turkey. The study design included eight randomized controlled trials (RCTs) [23–30], seven quasi-RCT [31–37], and thirteen observational studies [38–50].

**Table 1:** Characters of included studies.

Author	Year	Country	Location or data source	Study design	Sample size(NHD)	Disease status	Mean age (year)	Male (%)	Mean duration of dialysis (mo)*	Dialysis session		Reported outcomes
										NHD	CHD	
Chan[38]	2002	Canada	Toronto General Hospital	Observation cohort study	41(28)	ESRD(end-stage renal disease)	47(11)	N/A	NHD: 3.4Y; CHD: 2.8Y	8–10hours, every night	4hours, 3 times/week	LVHI, BP, Hb.
Friedman[39]	2002	Canada	Humber River Regional Hospital	Cross-sectional cohort study	54(23)	ESRD	44(20–65)	63.0%	NHD: 100(83)M; CHD: 29(17)M	6–7nights/week	3 times/week	Albumin
Heidenheim[31]	2003	Canada	London(Canada) Health Sciences Centre	Prospective nonrandomized(controlled) study	45(12)	ESRD	N/A	N/A	18M	6 nights/week	3 times/week	QOL;
Nesrallah[32]	2003	Canada	London(Canada) Health Sciences Centre	Prospective nonrandomized(controlled) study	43(12)	ESRD	N/A	N/A	18M	6 nights/week	3 times/week	BP; Drug usage
Pierratos[40]	2004	Canada	Humber River Regional Hospital	Retrospective study	88	ESRD	49(11)	65.0%	30(27)M	3–4nights/week	-	QOL; LVH;
Lindsay[33]	2004	Canada	London(Canada) Health Sciences Centre	Prospective controlled study	45(12)	ESRD	46.7(10.5)(28–76)	67.0%	5–36M	5–6 nights/week	3 times/week	BP; Mortality;
Schwartz[41]	2005	Canada	Humber River Regional Hospital	Retrospective cohort study	95(63)	ESRD	49.7(5.7)	68.0%	12M	5–6 nights/week	3 times/week	Hb; Drug usage
Culleton[23]	2007	Canada	University of Calgary and University of Alberta	Randomized Controlled study	52(26)	ESRD	54.1(12.8)	62.7%	6M	6 nights/week	3 times/week	LVH; QOL; BP; Drug usage
Johansen[42]	2009	U.S	United States Renal Data System database	Observation cohort study	1034(94)	ESRD	46.7(17.4)	65.9%	36M	5–6 nights/week	3 times/week	Mortality; Hospitalization
Manns[24]	2009	Canada	University of Calgary and University of Alberta	Randomized Controlled study	51(26)	ESRD	54.1(12.8)	62.7%	6M	5–6 nights/week	3 times/week	QOL
Powell[43]	2009	U.K	Western Infirmary renal unit	Case-Controlled study	106(53)	ESRD	51.2(15.5)	74.5%	>12M	3 times/week	3 times/week	URR; HB; BP; Drug usage
van Eps[44]	2010	Australia	Princess Alexandra Hospital	Observation cohort study	235(63)	ESRD	56.5(15.1)	63.8%	12M	3.5–4 times/week	3 times/week	Side-effects; Mortality
Lacson[45]	2010	U.S	Fresenius Medical Care, North America	Case-Controlled study	15989(655)	ESRD	61.9(15)	53.6%	12M	3 times/week	3 times/week	Mortality; Hospitalization; QOL; BP
Walsh[25]	2010	Canada	University of Calgary and University of Alberta	Randomized Controlled study	51(26)	ESRD	54.1(12.8)	62.7%	6M	5–6 nights/week	3 times/week	Albumin;
Jin[34]	2011	China	Second Military Medical University Changzheng Hospital	Nonrandomized control study	90(32)	ESRD	45(10.8)	91.0%	12M	3 nights/week	3 times/week	BP; LVHI;
Rocco[26]	2011	U.S	Frequent Hemodialysis Network (FHN) Trial Group	Randomized Controlled study	87(45)	ESRD	52.8(13.6)	65.5%	12M	6 nights/week	3 times/week	Mortality; LVH; BP; Hospitalization
Ok[35]	2011	Turkey	Long Dialysis Study Group	Prospective controlled study	494(247)	ESRD	45.5(13.4)	68.1%	12M	3 nights/week	3 times/week	Mortality; hospitalization; BP; Side-effect
Overgaard[46]	2011	Canada	Toronto, Ontario	Retrospective study	19(8)	ESRD	52(27–68)	N/A	31M	6 nights/week	3 times/week	BP
Rocco[27]	2011	U.S	Frequent Hemodialysis Network (FHN) Trial Group	Two separate randomized study	332(87)	ESRD	50.4(13.9)	62.0%	12M	6 nights/week	3 times/week	Mortality; LVH; QOL
Chan[28]	2012	Canada	Frequent Hemodialysis Network (FHN) Trial Group	Randomized Controlled study	87(45)	ESRD	52.8(13.6)	65.5%	12M	6 nights/week	3 times/week	LVM;
Demirci[36]	2012	Turkey	Long Dialysis Study Group	Prospective controlled study	120(60)	ESRD	49(11)	69.2%	12M	3 nights/week	3 times/week	BP, LVH;
Jin[37]	2012	China	Second Military Medical University Changzheng Hospital	Nonrandomized control study	90(32)	ESRD	45(10.8)	91.0%	12M	3 nights/week	3 times/week	BP; Hemoglobin;
Lacson[47]	2012	Canada	Fresenius Medical Care, North America	Observation cohort study	2808(746)	ESRD	53.8(14.2)	66.3%	24M	3 times/week	3 times/week	Mortality; PB; Albumin; Hemoglobin;
Chan[29]	2013	Canada	Frequent Hemodialysis Network (FHN) Trial Group	Randomized Controlled study	87(45)	ESRD	52.8(13.7)	65.5%	12M	6 nights/week	3 times/week	LVH;
Demirci[48]	2013	Turkey	Long Dialysis Study Group	Prospective cohort study	112(57)	ESRD	48(11.8)	70.5%	12M	3 nights/week	3 times/week	BP; Albumin; Hemoglobin
Overgaard[49]	2013	Canada	Toronto, Ontario	Retrospective study	12(6)	ESRD	51(27–66)	N/A	31M	6 nights/week	3 times/week	-
Kotanko[30]	2015	U.S	Frequent Hemodialysis Network (FHN) Trial Group	Randomized Controlled study	87(45)	ESRD	52.8(13.7)	65.5%	12M	6 nights/week	3 times/week	BP; Drug usage
Wald[50]	2015	Canada	St Michael's Hospital and St Paul's Hospital	Prospective cohort study	67(37)	ESRD	53.8(12.2)	55.2%	12M	3 nights/week	3 times/week	LVH; Haemoglobin; BP; Drug usage

Abbreviation: NHD: nocturnal hemodialysis; ESRD: End-stage renal disease; LVH: left ventricular hypertrophy; LVHI: left ventricular hypertrophy index; BP: blood pressure; QOL: quality of life; Hb; hemoglobin; URR: Urea reduction ratio.

*: Y: year; M: month. N/A: not available

**Fig 2:**
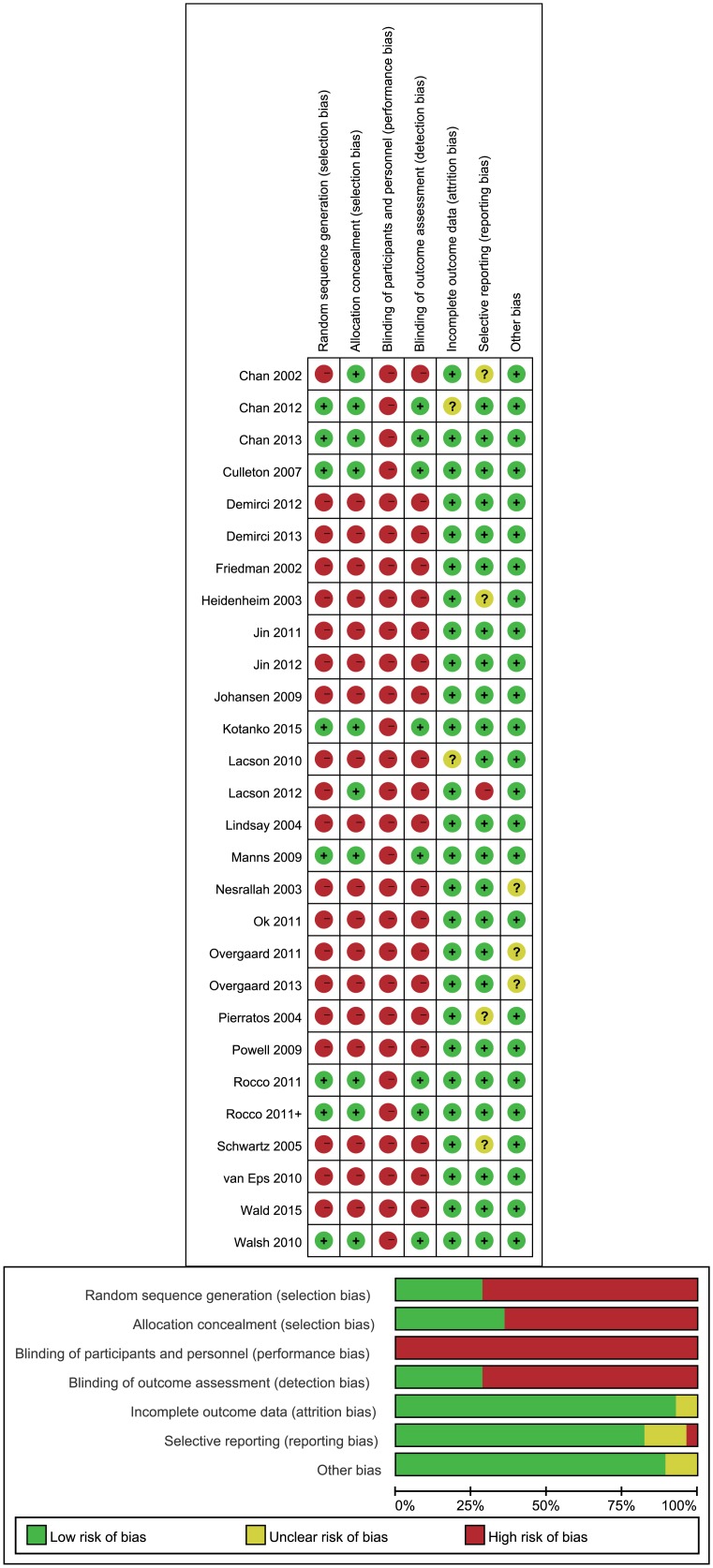
Methodological quality of trials included in the meta-analysis. Risk of bias graph and summary.

A total number of 22,508 ESRD patients were examined. The average reported age of patients was between 40–60 years while two studies did not mention the patients’ ages [31, 32]. The number of men was slightly greater than the number of women. The follow-up time duration was 6 months to 36 months. The schedule for NHD was 3 nights/week or 6–7 nights/week, and 3 times/week for CHD. The summary graph of risk of bias for each study is shown in Fig 2.

In our meta-analysis, mortality results were not significantly different between the NHD group and the CHD group (OR: 0.75; 95%CI: 0.52 to 1.10; p = 0.145). For number of hospitalizations, the CHD group had significantly fewer than NHD group (OR: 1.54; 95%CI: 1.32 to 1.79; p<0.001); in addition, there was no significant difference between the two groups in the number of infection hospitalizations (OR: 1.60; 95%CI: 0.48 to 5.35; p = 0.445).

Within cardiovascular-related variables, left ventricular hypertrophy (LVH, unit: g) and its index (LVHI, unit: g/m^2^) results both indicate the NHD group has significantly fewer occurrences than the CHD group (LVH: SMD: -0.39; 95%CI: -0.68 to -0.10; p = 0.009, LVHI: SMD: -0.64; 95%CI: -0.83 to -0.46; p<0.001). For the control of blood pressure, systolic blood pressure results also show the NHD group is significantly better than the CHD group (Random model: SMD: -0.33; 95%CI: -0.49 to -0.18; p<0.001, Fixed model: SMD: -0.17; 95%CI: -0.24 to -0.1; p<0.001). The Diastolic blood pressure index also shows the NHD group is significantly better than the CHD group (Diastolic blood pressure: SMD: -.032; 95%CI: -0.48 to -0.15; p<0.001, Mean arterial pressure: SMD: -0.69; 95%CI: -1.19 to -0.19; p = 0.007, Pulse pressure: SMD: -0.43; 95%CI: -0.75 to -0.12; p = 0.007).

For uremia-related variables, the concentration of serum albumin of the NHD group was significantly greater than the CHD group (SMD: 0.89; 95%CI: 0.41 to 1.36; p<0.001); the concentration of serum hemoglobin of the NHD group was also significantly greater than the CHD group (SMD: 0.42; 95%CI: 0.05 to 0.78; p = 0.025). The urea clearance index in the NHD group was significantly higher than the CHD group (SMD: 2.61; 95%CI: 1.76 to 3.46; p<0.001), and urea reduction ratio was also better in the NHD group (SMD: 1.39; 95%CI: 0.49 to 2.30; p = 0.003).

For the assessment of quality of life (QOL), NHD treatment only had significantly improved results for the patient in the SF36-Physical Components Summary (SMD: 0.43; 95%CI: 0.26 to 0.60; p<0.001). The results of the European QOL (SMD: -0.34; 95%CI: -1.83 to 1.14; p = 0.651) and the SF36-Mental Components Summary (SMD: 0.11; 95%CI: -0.07 to 0.28; p = 0.226) showed no significant difference between groups. In the patients’ drug usage assessment, the anti-hypertensive drug dosage in the NHD group was significantly lower than in the CHD group after dialysis (SMD: -0.48; 95%CI: -0.91 to -0.05; p = 0.005). However, the dosage of EPO was not different between groups (SMD: -0.23; 95%CI: -0.60 to 0.14; p = 0.222). In our assessment of the side effects of dialysis, the bacteremia (OR: 1.89; 95%CI: 0.96 to 3.74; p = 0.067) and septic (OR: 2.58; 95%CI: 0.73 to 9.16; p = 0.141) both showed no difference between groups.

Performing subgroup analysis, it was found that treatment with nocturnal dialysis 3 times/week yielded a significantly lower mortality rate than the control group (OR: 0.56; 95%CI: 0.34 to 0.92; p = 0.021; I^2^ = 74.8%), while the use of dialysis >3times/week yielded no significant differences (OR: 1.47; 95%CI: 0.68 to 3.19; p = 0.334; I^2^ = 30.6%). Through subgroup analysis of study designs it was discovered that randomized controlled trials and non-randomized controlled trials showed no significant differences in results (RCTs: OR: 0.98; 95%CI: 0.29 to 3.34; p = 0.977; Non-RCTs: OR: 0.73; 95%CI: 0.48 to 1.11; p = 0.140) (Table 2). Only in non-RCT researches, haemoglobin concentration showed significant difference between nocturnal dialysis and control group (SMD: 0.49; 95%CI: 0.10 to 0.88; p = 0.013). In the drug usage assessment, anti-hypertensive drug dosage in patients received more than 3 times per week nocturnal hemodialysis subgroup was significant less than CHD group (SMD: -0.64; 95%CI: -0.92 to -0.37; p<0.001), and in RCT design studies the anti-hypertensive drug dosage in the NHD group was significantly lower than in the CHD group (SMD: -0.64; 95%CI: -0.92 to -0.37; p<0.001). In subgroup analysis, the EPO dosage of 3 times/week subgroup showed significant less than CHD group (SMD: -0.45; 95%CI: -0.83 to -0.06; p = 0.022). However, the heterogeneity was not obviously reduced in all subgroup analysis.

**Table 2:** Subgroup analysis of nocturnal and conventional hemodialysis on ESRD patients.

Outcome	Subgroup	No. of trials	OR/SMD	LCI	UCI	p value	Heterogeneity	p for Heterogeneity
Mortality	>3 night/week	5	1.47	0.68	3.19	0.334	30.60%	0.217
	3 night/week	6	0.56	0.34	0.92	0.021	74.80%	0.001
	Randomized design	3	0.98	0.29	3.34	0.977	0%	0.552
	Nonrandomized design	8	0.73	0.48	1.11	0.14	73.10%	0.001
Systolic blood pressure	>3 night/week	4	-0.48	-0.71	-0.25	< 0.001	0%	0.911
	3 night/week	6	-0.27	-0.44	-0.09	0.003	47.20%	0.092
	Randomized design	3	-0.47	-0.71	-0.22	< 0.001	0%	0.086
	Nonrandomized design	7	-0.29	-0.46	-0.11	0.001	45.90%	0.803
Albumin	>3 night/week	1	7.26	5.77	8.76	< 0.001	-	-
	3 night/week	5	0.4	0.21	0.59	< 0.001	67.70%	0.015
Haemoglobin	>3 night/week	3	1.2	-1.38	3.77	0.363	98%	< 0.001
	3 night/week	7	0.17	-0.013	0.36	0.068	70%	0.003
	Randomized design	1	-0.3	-0.85	0.26	0.293	-	-
	Nonrandomized design	9	0.49	0.1	0.88	0.013	94%	< 0.001
Urea clearance index	>3 night/week	2	7.12	-1.97	16.21	0.125	97.20%	< 0.001
	3 night/week	3	1.83	1.05	2.61	< 0.001	93.90%	< 0.001
Anti-blood pressure drug	>3 night/week	2	-0.64	-0.92	-0.37	< 0.001	0%	0.807
	3 night/week	2	-0.32	-1.23	0.6	0.498	88.10%	0.004
	Randomized design	2	-0.64	-0.92	-0.37	< 0.001	0%	0.807
	Nonrandomized design	2	-0.32	-1.23	0.6	0.498	88.10%	0.004
EPO usage	>3 night/week	4	0	-0.75	0.75	0.994	86.30%	< 0.001
	3 night/week	3	-0.45	-0.83	-0.06	0.022	74.80%	0.019
	Randomized design	1	0.18	-0.27	0.63	0.434	-	-
	Nonrandomized design	6	-0.3	-0.7	0.09	0.132	81.50%	< 0.001

Abbreviations: ESRD: End-stage Renal Disease; OR: Odds ratio; SMD: Standard Mean Difference; LCI: Lower Confidence interval; UCI: Upper Confidence interval.

There was publication bias was found in systolic blood pressure results (Table 3, Begg’test, p = 0.592; Egger’s test, p = 0.001). However, no other publication bias was found. After correction of the results with “Trim and Fill” method the conclusion was not changed.

**Table 3:** Results of treatment effects of NHD versus CHD on end-stage renal failure patients.

Outcomes	No. of trials	Effect size	Value	LCI	UCI	P value	Heterogeneity	P for Heterogeneity	Model	Begg’s test	Egger’s test	Favors
Mortality												
Mortality	11	OR	0.75	0.52	1.1	0.145	63.40%	0.002	Random	0.533	0.87	Equal
Hospitalization												
Number of Hospitalization	2	OR	1.54	1.32	1.79	<0.001	0%	0.549	Fixed	-	-	CHD group
Number of Infection hospitalization	1	OR	1.6	0.48	5.35	0.445	-	-		-	-	Equal
Cardiovascular-associated variables												
Left ventricular hypertrophy (g)	3	SMD	-0.39	-0.68	-0.1	0.009	0%	0.74	Fixed	1	0.874	NHD group
Left ventricular hypertrophy index(g/m2)	5	SMD	-0.64	-0.83	-0.46	<0.001	0%	0.837	Fixed	0.806	0.669	NHD group
Systolic blood pressure	10	SMD	-0.33	-0.49	-0.18	<0.001	48.50%	0.042	Random	0.592	0.001	NHD group
			-0.17	-0.24	-0.1	<0.001	48.50%	0.042	Fixed	0.592	0.001	
Diastolic blood pressure	7	SMD	-0.32	-0.48	-0.15	<0.001	0%	0.967	Fixed	0.368	0.295	NHD group
Mean arterial pressure	2	SMD	-0.69	-1.19	-0.19	0.007	0%	0.646	Fixed	-	-	NHD group
Pluse pressure	2	SMD	-0.43	-0.75	-0.12	0.007	0%	0.326	Fixed	-	-	NHD group
Uremia-associated variables												
Albumin	6	SMD	0.89	0.41	1.36	<0.001	94.70%	<0.001	Random	0.133	0.186	NHD group
Haemoglobin	10	SMD	0.42	0.05	0.78	0.025	93.40%	<0.001	Random	0.721	0.248	NHD group
Urea clearance index	5	SMD	2.61	1.76	3.46	<0.001	94.60%	<0.001	Random	0.462	0.757	NHD group
Urea Reduction ratio(%)	3	SMD	1.39	0.49	2.3	0.003	91.60%	<0.001	Random	1	0.698	NHD group
QOL												
European Quality of life	2	SMD	-0.34	-1.83	1.14	0.651	92.30%	<0.001	Random	-	-	Equal
SF36(Mental Components Summary)	2	SMD	0.11	-0.07	0.28		0%	0.605	Fixed	-	-	Equal
SF36(Physical Components Summary)	2	SMD	0.429	0.258	0.6	<0.001	32.50%	0.224	Fixed	-	-	NHD group
Drug usage												
Anti-blood pressure drug	4	SMD	-0.48	-0.91	-0.05	0.03	76.60%	0.005	Random	0.734	0.585	NHD group
EPO usage	7	SMD	-0.23	-0.6	0.14	0.222	82.20%	<0.001	Random	0.23	0.302	Equal
Side Effect												
Bacteremia	2	OR	1.89	0.96	3.74	0.067	4.10%	0.307	Fixed	-	-	Equal
Septic	2	OR	2.58	0.73	9.16	0.141	85.80%	0.008	Random	-	-	Equal

Abbreviation: SMD: Standardized Mean Difference; OR: odds ratio; LCI: Lower confidence interval; UCI: Upper confidence interval; NHD: Nocturnal Hemodialysis; CHD: Conventional Hemodialysis

## Discussions

In this review, we analyzed the effects of NHD versus CHD in the treatment of ESRD. Our analysis included 28 trials with 22,508 patients. Our results demonstrate that NHD and CHD are similar in mortality and side-effects, and that NHD is superior to CHD in cardiovascular-associated and uremia-associated markers and in QOL and drug usage. CHD is relatively better than NHD for number of hospitalizations. In general, NHD has more advantages in clinical applications for ESRD patients.

In previously published meta-analyses, the results assessment was not comprehensive. Hui MJ et al. studied the effects of long-time dialysis in daytime or nighttime on survival rate compared to that of conventional hemodialysis [16]. Results showed that the survival rate of patients using prolonged hemodialysis was significant higher than those using conventional hemodialysis; however, residual confounders, which include the patients’ age, sex, presence of diabetes, and catheter use, interferes with the results in observational studies. This study included literatures with lower design quality while not having a comprehensive assessment index. Our research included more high quality design articles to find that nocturnal dialysis does not significant improve the mortality of patients; however, subgroup analysis of treatment 3times/week showed reduced mortality rates. This may be due to the fact that the study used patients with relatively mild uremic symptoms while further study is needed to draw conclusions for the specific causes. Julia Thumfart et al. evaluated the effect of intensified nocturnal hemodialysis on ESRD patients compared to conventional hemodialysis in 2014 [15]. That study found that intensified hemodialysis could significantly improve the patients’ blood pressure, uremia-associated variables, and psychosocial variables, and could reduce the usage of antihypertensive and phosphate binders. However, there was no assessment of patients’ mortality and QOL. Our research supports the evidence that intensified hemodialysis could improve cardiovascular-related and uremia-related indicators; we also defined that nocturnal dialysis could improve the patients’ QOL. Paweena Susantitaphong et al. assessed the effects of frequent nocturnal hemodialysis on ESRD patients using the indicators of left ventricular mass and cardiovascular mortality in 2012 [14]. Unfortunately, this research had a paucity of randomized controlled trials. The results supported that frequent or extend hemodialysis could improve cardiac morphology and function; however the outcome of long-term clinical application was limited. Our study includes a longer follow-up period of up to 36 months and RCTs. Our results support the above conclusion and consider long-time nocturnal hemodialysis as beneficial for cardiovascular and uremia-related indicators.

It is very common for cardiovascular complications to occur in long-term hemodialysis patients, including hypertension, coronary heart disease, arrhythmia, and heart failure. Cardiac vascular disease events like cerebrovascular accident, ischemic heart disease, congestive cardiac failure, peripheral vascular disease are also much more prevalent in the chronic kidney disease population. Furthermore, cardiovascular complications are the most common causes of death in ESRD patients and the mortality rate for dialysis patients is up to 10–30 times higher than the matched population [51]. The high mortality indicates the effect of drugs to reduce the incident of cardiovascular disease is not ideal. Therefore, researchers presume the incidence of cardiovascular disease in dialysis patients may have a special pathophysiological process.

There are two parallel factors which may contribute to cardiovascular disease in ESRD patients. The first is a change of cardiac morphological including LVH and left ventricular (LV) dysfunction caused by mechanical or hemodynamic overload and the second being the change of vasculature including atherosclerosis and vascular calcification. These two factors can eventually result in cardiomyopathy and arterial thrombosis [52]. Uremia-related hyperphosphatemia, high calcium and phosphorus deposition, and hyperparathyroidism may be the direct reason for vascular calcification in ESRD patients. Currently it is popular to assess the patient’s dialysis schedule with cardiovascular-related symptoms, in which left ventricular hypertrophy is an important predictor of cardiovascular side effects. Thus, many RCTs use left ventricular mass (LVM) as the primary outcome [23, 28]. Our results show nocturnal dialysis have positive effects on the prevention of cardiovascular disease, which can enhance blood pressure control and reduce serum phosphate, hence reducing the risk of cardiovascular disease.

Although our study shows nocturnal dialysis has a great positive effect on ESRD patients, this approach also has a higher failure rate. For example, a 12 month follow-up period study pointed out that the technique’s survival rate is 79.2% [45]; a 24 months follow-up period study showed the technique’s survival rate by then was only 24.93% [47], meaning about 3/4 of ESRD patients were unable to continue nocturnal hemodialysis treatment. These studies found that the reasons of technique failure included infection, catheter dysfunction, and psychosocial problems in the early stage and ultrafiltration -failure and catheter-related infection in later stages. Therefore further research is needed to look into ways of increasing the technique’s survival rate on patients with high frequency nocturnal dialysis by improving technology and reducing complications.

We comprehensively evaluated the outcome measurements of nocturnal dialysis for ESRD, but still our study had several limitations. First, we did not have specific individual data for all the trials and thus our statistical approach was done at a study level. Second, the quality of included trials was relatively low, although this review included many outcome measures, single measure conclusions were considered from small sample studies of low quality. Third, there was heterogeneity in several outcomes among included trials. Finally, we were not able to use subgroup analysis or meta-regression to reduce the heterogeneity because there was a lack of trials using a single medicine.

Nocturnal hemodialysis and conventional hemodialysis perform similarly in ESRD patients’ mortality and side-effects. In cardiovascular-associated and uremia-associated results NHD is superior to CHD; and in QOL and drug usage NHD intervention is relatively better than CHD. For number of hospitalizations, CHD was relatively better than NHD. In general, NHD has more advantages in clinical application for ESRD patients.

## Supporting Information

S1 Search Strategy (DOCX)
