# Supplementary material for: Effect of Nocturnal Hemodialysis versus Conventional Hemodialysis on End-Stage Renal Disease: A Meta-Analysis and Systematic Review
Source: PLoS One. 2017 Jan 20;12(1):e0169203. doi: 10.1371/journal.pone.0169203 (PMC5249197; doi:10.1371/journal.pone.0169203)
Supplement: S1 Search Strategy — (DOCX) [file pone.0169203.s001.docx]

**Search Strategy:**

| **Medline** | **Search strategy** |
| --- | --- |
| **#1** | ("Hemodiafiltration"[Mesh]) OR "Renal Dialysis"[Mesh] |
| **#2** | dialysis OR haemodialysis OR hemodialysis OR “extracorporeal blood cleansing” OR hemodialyse OR hemorenodialysis OR hemotrialysate |
| **#3** | #1 OR #2 |
| **#4** | "Kidney Failure, Chronic"[Mesh] |
| **#5** | “end stage kidney disease” OR “end stage kidney failure” OR “end stage renal dysfunction” OR “end stage renal failure” OR “end stage renal impairment” OR “end stage renal insufficiency” OR “end-stage kidney disease” OR “end-stage kidney failure” OR “end-stage renal disease” OR ESRD OR “stage 5 kidney disease” OR “stage 5 renal disease” |
| **#6** | #4 OR #5 |
| **#7** | nocturnal OR night OR evening OR “extended hours” |
| **#8** | #3 AND #6 AND #7 |

| **Embase** | **Search strategy** |
| --- | --- |
| **#1** | 'hemodialysis'/exp OR dialysis OR haemodialysis OR hemodialysis OR 'extracorporeal blood cleansing' OR hemodialyse OR hemorenodialysis OR hemotrialysate |
| **#2** | 'end stage renal disease'/exp OR 'end stage kidney disease' OR 'end stage kidney failure' OR 'end stage renal dysfunction' OR 'end stage renal failure' OR 'end stage renal impairment' OR 'end stage renal insufficiency' OR 'end-stage kidney disease' OR 'end-stage kidney failure' OR 'end-stage renal disease' OR esrd OR 'stage 5 kidney disease' OR 'stage 5 renal disease' |
| **#3** | nocturnal OR night OR evening OR 'extended hours' |
| **#4** | #1 and #2 and #3 |

| **Cochrane** | **Search strategy** |
| --- | --- |
| **#1** | MeSH descriptor: [Kidney Failure, Chronic] explode all trees |
| **#2** | MeSH descriptor: [Renal Dialysis] explode all trees |
| **#3** | #2 or dialysis or haemodialysis or hemodialysis or 'extracorporeal blood cleansing' or hemodialyse or hemorenodialysis or hemotrialysate |
| **#4** | #1 or "end stage kidney disease" or "end stage kidney failure" or "end stage renal dysfunction" or "end stage renal failure" or "end stage renal impairment" or "end stage renal insufficiency" or "end-stage kidney disease" or "end-stage kidney failure" or "end-stage renal disease" or ESRD or "stage 5 kidney disease" or "stage 5 renal disease" |
| **#5** | nocturnal or night or evening or "extended hours" |
| **#6** | #3 and #4 and #5 |
